# Behind the Scenes of a Technologically Enhanced Intervention for Caregivers of People With Dementia: Protocol for a Feasibility and Acceptability Study

**DOI:** 10.2196/42655

**Published:** 2023-03-31

**Authors:** Abigail Gómez-Morales, David Coon, Rodney P Joseph, Teri Pipe

**Affiliations:** 1 Edson College of Nursing and Health Innovation Arizona State University Phoenix, AZ United States

**Keywords:** Alzheimer's disease, dementia, virtual reality, information and communication technology, ICT, caregivers, pilot study

## Abstract

**Background:**

Alzheimer's disease affects 55 million people worldwide. As the disease progresses, these individuals require a devoted caregiver, often a family member, who provides evolving complex care. Caregivers can experience a variety of ongoing stressors, resulting in reductions in caregiver emotional well-being (and other quality-of-life indicators). Information and communication technologies provide an excellent opportunity to train caregivers remotely and help them to manage these stressors and related distress.

**Objective:**

This protocol describes the theoretical rationale, study design, and methods of a new, technologically enhanced psychoeducational skill-building intervention for caregivers of people with dementia that includes a virtual reality component.

**Methods:**

*Through Alzheimer’s Eyes* is a 4-week, single-arm, pre-post test pilot study consisting of 4 sessions of 90 minutes each that are delivered by videoconferencing. These sessions include a weekly virtual reality experience characterizing the journey of an older Latina with Alzheimer's disease from her perspective to help caregivers see through the eyes of a person with dementia. The 4 sessions cover the skill-training topics of communication, managing challenging behaviors and unhelpful thoughts, the importance of self-care, and mindfulness—all of which are key components designed to reduce stress and distress in family caregivers. Individual interviews conducted before and after the intervention gather participant insights into the intervention, evaluate its feasibility and acceptability, and assess its impact on key outcomes.

**Results:**

Data collection for the study started in January 2022, and the results are expected to be submitted for publication in the second half of 2023. Twenty caregivers from the United States have completed the workshop to date. Preliminary data gathered from these participants support the intervention’s feasibility and acceptability.

**Conclusions:**

*Through Alzheimer’s Eyes* leverages existing technology combined with psychoeducational skill building to help caregivers manage their stress, regardless of their location.

**International Registered Report Identifier (IRRID):**

DERR1-10.2196/42655

## Introduction

### Background

Alzheimer's disease and related dementias (ADRD) are progressive diseases that primarily affect individuals aged ≥65 years. Worldwide, these diseases affect approximately 55 million people [1]; in the United States, 6.5 million people currently live with ADRD. Given the nature of ADRD, devoted family caregivers provide evolving complex care during the disease trajectory, which can amount to as much as 80% to 100% of the care [2]. This constant care translates into ongoing stressors for caregivers because they must ensure their loved one’s well-being and quality of life. Caregiver tasks include, but are not limited to, aiding the loved one in activities of daily living and instrumental activities of daily living such as bathing, dressing, eating, managing medications, managing problematic behaviors, handling finances, attending physician appointments, and shopping [3]. Caring for people with ADRD can be further complicated by the caregiver’s own physical health limitations, mental health concerns, and other stressors (eg, from being housebound to functional impairments, history of depression or anxiety, financial concerns, and employment-related challenges) [3,4]. In addition, the stress associated with family caregiving for loved ones with dementia often translates into deterioration of caregivers’ physical and mental health [3,5-8], whether or not caregivers face substantive vulnerabilities or life challenges beyond their caregiver role.

Numerous in-person interventions for caregivers of people with ADRD are available that promote aging in place, caregiver self-care, caregiver burden, and skills training while improving quality of life, relationships, and stress and distress management skills [4,5,9]. A growing number of these interventions have shown effectiveness for decreasing burden, depression, and stress, as well as improving quality of life and one’s sense of competence [10,11]. However, in-person interventions present many limitations, especially for caregivers who have scheduling conflicts, work full-time or part-time, live in remote locations, have limited access to transportation, or have a limiting illness [12,13].

Caregiving interventions delivered through information and communication technologies (ICTs) can overcome many of the constraints of in-person intervention delivery and can play a crucial role in reaching out to caregivers of people with ADRD. ICTs are tools and procedures used to acquire and distribute information and enhance communication via technologies such as hardware, software, or the internet. ICTs enable the collection, capture, processing, production, treatment, registry, storing, exchanging, and presentation of information [14,15]. Regardless of their age, many caregivers are willing and eager to incorporate new technological tools into their daily lives [16,17]. However, there are limited studies in the literature that have used ICTs to deliver caregiving interventions to caregivers of people with ADRD. Among the few interventions that have done so, delivery channels have included virtual reality (VR), assistive devices, and internet-based interventions, and all have met with high acceptance [18,19]. Of note, VR is a computer-created interaction-enabled simulation that allows the user to engage in a realistic environment through a specific VR headset [19].

ICT interventions have been successful in training formal (ie, paid professionals and staff) and informal caregivers. Examples of studies include qualitative projects with formal and informal caregivers. One study indicated that the dementia simulation program helped participants to have more empathy for people with dementia as well as enhanced understanding of dementia and risks of developing dementia, to increase their awareness of any changes in the attention and affection they give to people with dementia, and to acquire and reinforce new care strategies and change other strategies such as communication techniques [20]. Another qualitative study with health care providers also highlights that simulation of what it is to have dementia is an insightful, evocative, and convincing immersive experience that takes dementia awareness training to the next level [21]. When the general population interacts with the VR simulation of a person with dementia, results indicate that after the experience, the participants’ sense of community is increased. The subscales of the Sense of Community Scale—solidarity and proactiveness as well as reliance on others—also showed a statistically significant positive shift. In addition, participants changed their attitudes toward dementia after the educational program [22].

Two studies are important to highlight when considering projects that use VR to train informal caregivers of people with dementia:

1. The program *Into D’mentia* consists of a 20-minute simulated experience for caregiver participants that focuses on the beginning stages of dementia (primarily of the Alzheimer's disease type). The program helps caregivers to feel the confusion that a person with ADRD might experience when their routine activities cannot be completed as desired. The simulation was followed by a one-on-one conversation with the trainer and a half-day group meeting with other participants. No statistically significant differences were found on key outcomes (eg, empathy, sense of competence, quality of relationship with care recipient, depression, anxiety, and caregiver burden). However, caregivers did report a heightened understanding of dementia and that, after the program, they changed the way they cared for their loved ones. Study limitations delineated by the researchers include the minimal number of simulation and group sessions and a lack of applicability to caregivers whose loved ones have progressed beyond the early stages of dementia [11,23].
2. The *Through the D’mentia Lens 360º* program is a VR adaptation of *Through the D’mentia Lens* scenarios. The experience consists of a 360-degree simulation movie and a self-paced e-course covering topics about dementia and communication. The results indicate that the simulation intervention was user friendly and improved understanding of dementia. Furthermore, the project contributed to improved caregiver empathy on the subscales perspective taking (Interpersonal Reactivity Index questionnaire), proactive competence when caring for a person with dementia as well as resilience (Trust in Own Abilities questionnaire), and more positive interactions with the person with dementia (Dyadic Relationship Scale). Study limitations included the lack of a control group as well as a small sample size and personal circumstances that might have affected the outcome of the study (eg, involvement of professional caregivers or deterioration of the person with dementia), testing effects, sampling bias owing to selection of particular informal caregivers recruited by case managers, using nonvalidated questionaries, and having a large proportion of female participants; in addition, the impact of each component of the intervention was not evaluated individually (rather, the intervention as a whole was evaluated) [24].

Thus, although there are new interventions using ICTs, including simulation or VR for training formal and informal caregivers of people with dementia, there is still a gap in how to implement and use new technologies to help educate caregivers about dementia and provide them with different skills to manage behavior challenges and reduce their own stress.

### Objectives

To address the paucity of ICT-delivered ADRD caregiving interventions and known limitations, such as the lack of more VR sessions and group meetings, we designed a user-friendly multicomponent ICT-delivered intervention entitled *Through Alzheimer’s Eyes*. This intervention is guided by social cognitive theory (SCT), owing to its relevance when learning in a social environment, and cognitive behavioral therapy (CBT) as a model to help caregivers develop and master appropriate skills. Several of the intervention’s components are drawn from prior caregiver intervention studies grounded in these theories conducted by Gallagher-Thompson et al [25], Coon et al [26], Belle [27], and Coon [28]. The intervention leverages effective design considerations of previous in-person ADRD caregiving interventions (group format, educational modules and handouts, discussions with participants, home assignments, and individual weekly sessions) to support the participant learning process and promote behavior change. Furthermore, the intervention is designed to prepare caregivers of people with ADRD for their role as caregivers in alleviating stress and distress while improving communication, behavioral management skills, and management of unhelpful thoughts, as well as well-being, with the support of VR. *Through Alzheimer’s Eyes* is delivered through the Zoom videoconferencing platform (Zoom Video Communications, Inc) and augmented with VR. In this paper, we describe the theoretical rationale, study design, and methods of a 4-week pilot study designed to evaluate the acceptability and feasibility of the intervention. The aims of the study were to (1) evaluate the feasibility and acceptability of our new ICT-delivered program for caregivers of people with ADRD focusing on VR, educational, and skill-building components; (2) examine patterns of change among key outcomes targeted by the intervention, including communication skills, management of behavioral issues, and management of unhelpful thoughts; and (3) learn about the benefits of the sessions from the participants’ perspective. Feasibility and acceptability will be assessed by examining recruitment strategies, participant retention, adherence to the intervention, and participant feedback provided through a postintervention satisfaction survey and one-on-one qualitative interviews. Pre-post intervention changes in key caregiving outcomes will be assessed using validated surveys.

## Methods

### Study Design and Participants

The study is a 4-week, single-arm, pre-post test pilot study designed to examine the feasibility and acceptability of a technologically enhanced skill-building intervention. The study is ongoing and to date has enrolled 20 adults who are caregivers of people with ADRD. The inclusion criteria are as follows: (1) self-report of being an unpaid primary caregiver of a person with ADRD for at least 6 months, (2) the caregiver and the person with ADRD must live together, (3) the caregiver must be aged ≥18 years, (4) they must be able to speak and read English, and (5) they must have access to a computer or similar device with a camera, speaker, microphone, and reliable bandwidth. Caregivers are not considered for the study if they have (1) vision or hearing impairment that impedes full participation over Zoom or (2) serious memory problems themselves. The research staff consults the principal investigator before determining eligibility if (1) the care recipient has been diagnosed with a severe mental illness before the age of 45 years (as reported by the caregiver), (2) the caregiver reports having memory loss, (3) the caregiver has frequent difficulty comprehending the questions during the screening, or (4) the caregiver is currently participating in a program, study, or workshop for caregivers designed to reduce stress and distress.

### Setting

As this is an ICT-delivered intervention, participants can join from any location in the United States. Every participant selects a private location that best meets their needs to connect to the intervention via videoconference. Researchers connect from their preferred location while ensuring privacy and confidentiality.

### Ethics Approval

The study protocol for *Through Alzheimer’s Eyes* was approved by the Arizona State University institutional review board (STUDY00014856).

### Informed Consent

Written informed consent was obtained after initial eligibility screening and before intervention delivery. The consent form contained detailed information about the purpose of the research; intervention details such as assessments to be performed, program schedule, time invested in the intervention, risks, privacy and confidentiality protection, what happens to the information collected for research, funding, cost, and payment (a total of US $25 in the form of eGift cards) for completion of the study’s interviews; whom to talk to should the participants have any questions; and a question asking participants whether they are willing to be contacted about participating in future studies (participants did not have to agree to participate in this study).

### Privacy and Confidentiality Protection

To ensure privacy and confidentiality, all the records are kept confidential and saved in password-protected secure servers, and any written documentation is kept in locked file cabinets in a secured access research office.

### Through Alzheimer’s Eyes Intervention

The 4-week intervention includes dementia education and skill-training components on techniques for communicating effectively, managing challenging behavior, controlling unhelpful thoughts, and caring for oneself, which are vital when providing person-centered care [18]. Participants engage with one another and their group leaders through activities facilitated by the group leaders, including mini-lectures, VR experiences, discussions, mindfulness exercises, and other relevant intervention activities, to master the skills. The facilitator has access to the VR set and plays the VR experience for the participants. Participants are divided into two groups: (1) immersive VR users and (2) semi-immersive VR users (those who are unable to use the VR headset owing to experiencing cybersickness while using the headset). Semi-immersive VR users will experience what life is like for a person with Alzheimer's disease through visualization on the user’s computer via screen sharing.

The semi-immersive VR experience facilitated by Embodied Labs [29] consists of visualizing the journey of “Beatriz,” an older Latina with Alzheimer's disease. Participants see through Beatriz’s eyes and experience what it is like to have memory challenges, hearing impairments, confusion, and hallucinations, among other limitations associated with the disease.

### Intervention Content

The selected topics for the psychoeducational skill-training components of the intervention sessions include not only increasing dementia knowledge but also improving the following skills: (1) communication with the loved one, family, friends, and health care providers; (2) managing challenging behavior; (3) controlling unhelpful thoughts; and (4) mindfulness and other relaxation skills. The goal is to help caregivers manage their most pressing concerns and sources of their stress successfully and understand the changes in the brain associated with Alzheimer's disease and the symptoms their loved one displays [9,30]. The topics were chosen based on existing literature about caregiving’s most pressing concerns and sources of stress, as well as past successful psychoeducational interventions such as *Coping with Caregiving* [25,26,31]. Communication, problem-solving, and combativeness (behavioral issues) are topics where caregivers perceive they need the most help. Focusing on skill-building activities with education about the disease helps caregivers manage and decrease their stress. In addition, caregivers often need assistance and reminders about maintaining a healthy lifestyle and relaxation [26,30-32].

Communication skills have been chosen because caregivers need to learn how to communicate effectively with a person with dementia. As the cognition of the person with dementia deteriorates, new ways of communication are encouraged to be implemented to ensure that the caregiver communicates effectively, and the care recipient understands the message. Communication education and skill-training was divided into (1) how to communicate assertively with family and others, as well as the differences between passive and aggressive communication; and (2) how to communicate with the loved one, including verbal and nonverbal tips to deliver messages to their loved ones as the disease progresses. Effective communication helps to increase caregiver and care recipient satisfaction [33,34], and participants from previous interventions considered communication an excellent coping skill [33].

Increasing caregiving skills in managing challenging behavior or combativeness helps the caregiver to provide better and more effective care. Helping the caregiver to find the root or trigger of the challenging behavior decreases their stress [25]. During the intervention, the caregiver learns about the triggers and how to resolve them by helping the care recipient to be calm and helping to decrease the frequency of challenging behavior. Managing these challenges can have a positive impact by improving the quality of the relationship between caregiver and care recipient [5,26].

Learning to control unhelpful thoughts has been chosen as a key skill to help the caregiver change these unhelpful thoughts into productive ones to improve their own well-being [5,25,26]. During the session, participants identify unhelpful thoughts and learn different ways to replace them with more helpful ways of thinking about their loved ones, themselves, and their caregiving situations. Finally, mindfulness skills help the caregiver to be aware of the present moment, such as being aware of unhelpful thoughts when they occur, and offer the opportunity to learn different relaxation techniques to help decrease stress [5,26,27]. The techniques include mindful breathing, mindful eating, and body scan, as well as access to the Calm app for more resources.

### Interventionists

A master’s prepared social worker and a Doctor of Philosophy candidate in nursing were trained to deliver the intervention under the supervision of the second author, an experienced caregiver intervention researcher and geropsychologist. The main role of the interventionists is to deliver the weekly intervention sessions, guide the weekly individual sessions with the participants, provide extra resources when needed, provide feedback related to the home exercises, and guide the discussions during the sessions.

### Intervention Sessions

The intervention will consist of 4 sessions delivered via videoconference (Zoom) over 4 weeks to a group of 5 to 9 family caregivers. Each group session lasts 90 minutes and consists of a new topic, a discussion that includes a review of the previous week’s topic, a VR experience, home practice, and mindfulness exercises. The participants join the interventionist individually for a 30-minute discussion to revisit content and work on personalized goals between group sessions. Textbox 1 illustrates the content of both group and individual sessions. Mini-lectures, VR experiences, discussions, mindfulness activities (including options for mindfulness apps), and home exercises or home practice provide excellent opportunities to reinforce the knowledge acquired during the group sessions and facilitate different discussions about the topics covered during these sessions.

**The intervention’s weekly modules, activities, and content.**

**Week 1**

- Weekly topic: learning about Alzheimer's disease, how it affects the brain and symptoms, and communication skills (to decrease challenging behavior)
- Virtual reality (VR) experience 1: focus on early stages of the disease
- Mindfulness: mindful breathing; introduction to mindfulness resources

**Week 2**

- Weekly topic: problem-solving, managing disruptive and challenging behavior, and applying communication skills
- VR experience 2: focus on middle stages of the disease
- Mindfulness: mindful eating

**Week 3**

- Weekly topic: controlling unhelpful thoughts; emphasis on actions that can lead the caregiver to have unhelpful thoughts, especially those related to quality of care and future options
- VR experience 3: focus on late stages of the disease
- Mindfulness: mindful walking

**Week 4**

- Weekly topic: importance of self-care and revision of the covered topics
- VR: experience all 3 VR experiences covering early, middle, and late stages of the disease
- Mindfulness: body scan
- Between sessions
- A 30-minute one-on-one Zoom session with an interventionist
- Review of the materials and work on individual stressors and goals

### VR Experience

**Figure 1:**
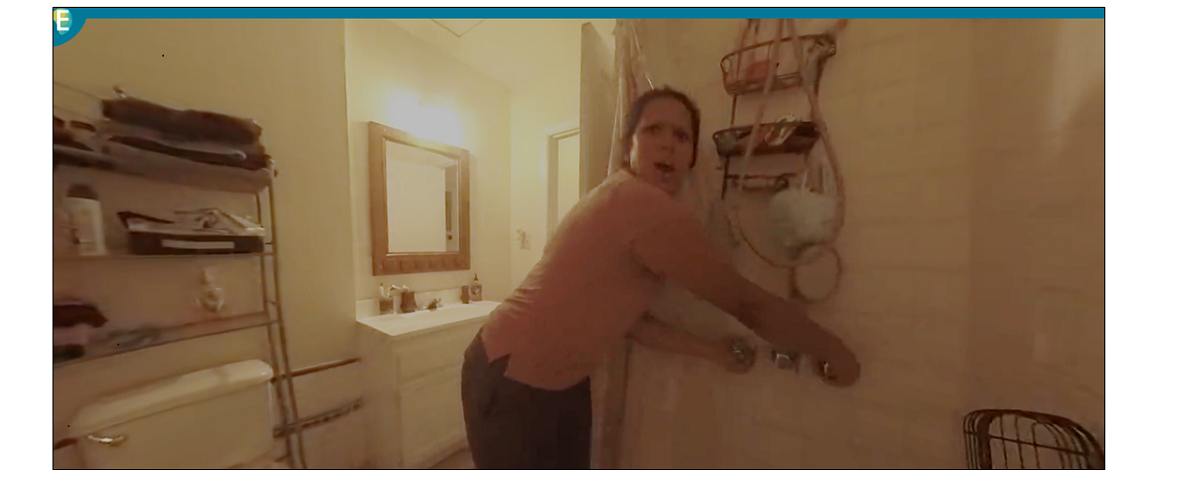
Screenshot of a virtual reality scene created by Embodied Labs (reproduced with permission). In this scene, the participant sees the bathroom through Beatriz’s eyes and perspective; Beatriz is in the middle stages of Alzheimer's disease. This scene corresponds to when she takes a bath. The participant is experiencing Beatriz’s ability to complete daily tasks.

Participants watch a different VR experience every week. The VR experience facilitated by Embodied Labs consists of 3 modules targeting 3 different stages of the disease: early, middle, and late. The experience portrays the story of Beatriz, an older Latina with Alzheimer's disease, and her journey from the early stages to the late stages of the disease, emphasizing her struggles with communication as visual and hearing impairments affect her activities of daily living and her family. The participant watches the video through the eyes of Beatriz (Figure 1).

### VR Equipment

**Figure 2:**
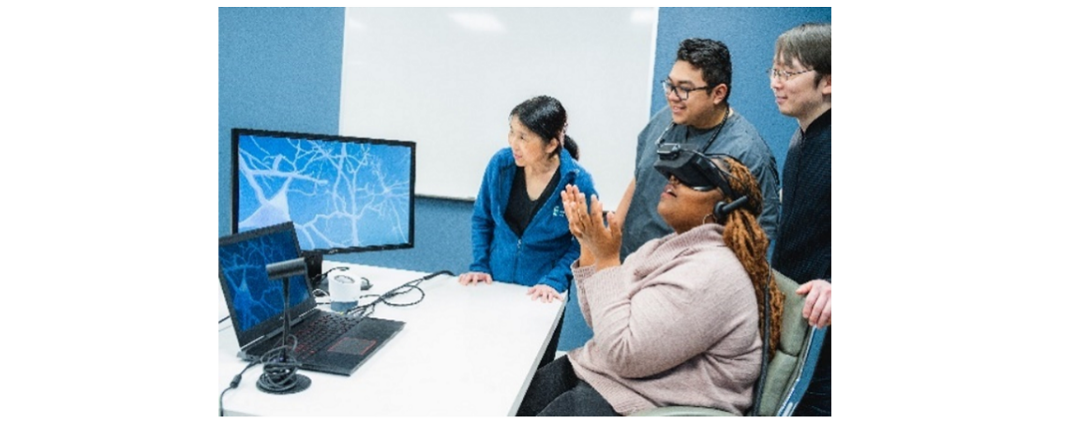
Set-up of the virtual reality equipment (reproduced with permission from Embodied Labs).

The VR equipment includes materials for the instructor as well as for the participants. The instructor VR headset (Figure 2) consists of a laptop computer with the software and a headset connected to the laptop computer. The equipment and software were purchased from Embodied Labs, whose representative provided one-on-one training on the VR software and use of the equipment before its implementation in the intervention. Both the equipment and the software are very user friendly.

The participants have a VR headset adapted for their mobile phones to visualize the experience from their homes. The mobile phone–adapted headset for the participants was bought from a web-based vendor, and the brands used included BNext and Viotek, depending on market availability.

### Theoretical Framework

**Figure 3:**
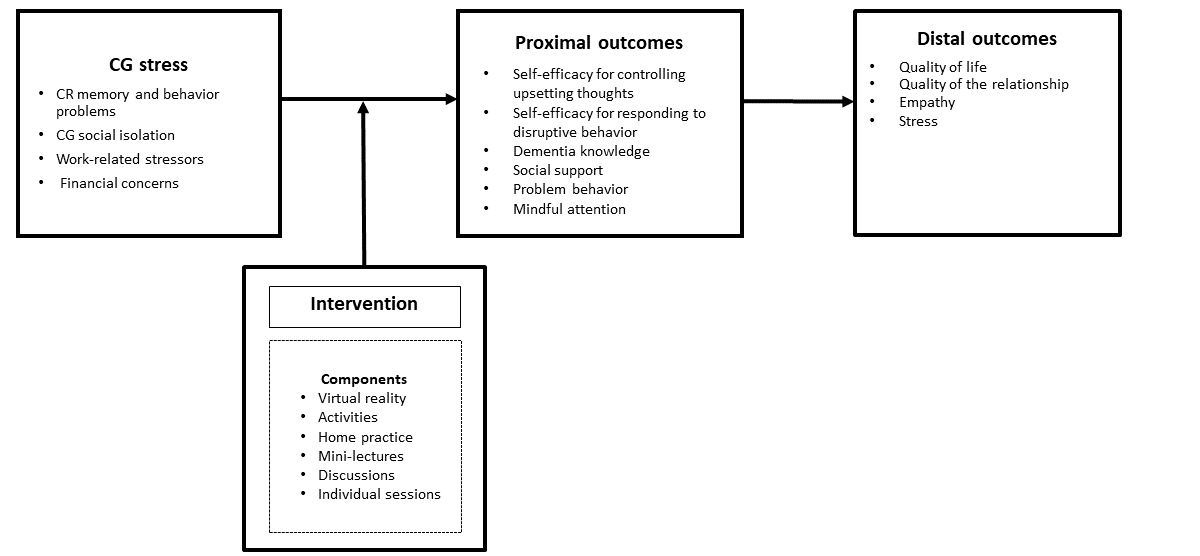
Theoretical framework. CG: caregiver; CR: care recipient.

The overall intervention is guided by two main frameworks: (1) SCT, which focuses on behavior change; and (2) CBT to help guide participant training in cognitive and behavioral skills (Figure 3).

SCT is a dynamic, interpersonal behavioral theory. SCT posits that the interaction among the beliefs and attitudes of the person, their behavior, and their social and physical environment influence each other reciprocally and dynamically. SCT explains that acquiring and maintaining new behavior is best achieved when people learn in a social environment while drawing on past experiences [35]. SCT constructs emphasize that learning comes from four sources: (1) mastery experience: acquiring knowledge through obtaining information, (2) vicarious experience: watching others perform an activity, (3) verbal persuasion: learning from feedback, and (4) emotional arousal: learning from past experiences [35,36]. SCT and CBT concepts and intervention components, definitions, relationships among the constructs, outcomes, and scales used to measure the outcomes are described and summarized in Multimedia Appendix 1.

The cognitive behavioral skill-building model (based on CBT) is a counseling approach focusing on thoughts and behavior that affect mood. One of the main applications of CBT concerns helping with mental health problems such as anxiety or depressive symptoms, where thoughts and behavior get in the way of a person’s mood and functioning [36-38]. CBT is a fundamental component in this intervention, guiding the interventionist to help the caregiver develop cognitive (thought monitoring and restructuring) and behavioral (problem-solving, behavior management, communication, and relaxation) skills [36,37,39].

### Feasibility and Acceptability Evaluation

Feasibility and acceptability will be evaluated by examining the different recruitment strategies, adherence to the intervention, and treatment implementation. In addition, individual postintervention Zoom interviews will provide further insight into the benefits and usefulness of the intervention.

### Adherence Strategies

To encourage participation over the 4 weeks, strategies include weekly reminders from one session to another, calendar invitations, reminders during the one-on-one videoconference calls, and reminders in emails exchanged during the week. At the end of the study, adherence will be examined by comparing completers with dropouts.

### Treatment Implementation

We have chosen the treatment implementation model developed by Burgio et al [40] to evaluate the feasibility and acceptability of the intervention. The treatment implementation model focuses on the 3 components of treatment delivery, receipt, and enactment to monitor activities in the different groups, minimize intervention efficacy bias, and evaluate treatment. The goal is to present the intervention as intended.

Treatment delivery focuses on the interventionists and the consistency of the intervention as delivered in the different groups [40,41]. Strategies to ensure consistency include manuals for the interventionists with a timeline to ensure that the same amount of time is devoted to the intervention activities across the different groups. In addition, a script is available to guide the individual Zoom calls. Weekly monitoring and feedback ensure that the interventionists deliver the sessions consistently.

Treatment receipt records whether the participant has received the intervention and understands the concepts as intended [40,41]. Strategies to evaluate the participant’s knowledge include interventionists asking the caregiver about using the different concepts during the individual weekly Zoom calls and whether the caregiver has any questions about the content covered. In addition, during the sessions, the interventionist asks the participants for examples and requests them to share some of the home practices. This measure ensures that participants understand the concepts discussed during the session. The postassessment survey and an individual postintervention Zoom interview also include questions about the caregiver’s perspectives of the intervention and their experiences.

Treatment enactment allows us to record and assess the use of skills and knowledge acquired during the different sessions in the participants’ daily activities [40,41]. Strategies include recording in the enactment form after each session whether participants completed some written home practice, participated in the discussions, displayed understanding of the content through the question-and-answer sessions, and demonstrated the use of skills in the session, as well as whether they talked about using the skills outside of the intervention sessions.

### Perception of Benefit

A 16-item postinterview survey asks participants about the perceived benefits derived from their involvement in the intervention. The survey is drawn from previous research [27,40] with family caregivers, with ratings ranging from *not at all* and *a little* to *a lot*. The survey questions include “Overall, how much do you think you benefited from participating in the program?” “How much did participation in the project help enhance your ability to care for your loved one?” “How much did participation in the program help to control upsetting thoughts?” The findings are expected to help inform whether the intervention meets caregiver needs.

### Perceived Changes

The survey on perception of change includes 8 questions [27,40] indicating self-perceived changes in several key areas, including mood, communication, confidence, level of preparation for future care, and physical health. The ratings range from *declined substantially*, *declined minimally*, and *stayed the same* to *improved minimally* and *improved substantially*. The survey questions include “Do you think the communication with others, besides your loved one, has improved, declined, or stayed the same?” “Do you feel your overall confidence that you can provide care for CR has improved, declined or stayed the same?” [27,40].

### Pre- and Postintervention Web-Based Surveys

To provide preliminary data on the impact of the intervention, the caregiver participants complete pre-post surveys to assess potential changes in proximal (eg, self-efficacy in responding to disruptive behavior and dementia knowledge) and distal (eg, quality of life and empathy) outcomes. The study’s intervention outcomes are assessed with the following scales: Revised Scale for Caregiving Self-Efficacy, Preparedness for Caregiving Scale, Problem-Solving Self-Efficacy Scale, Alzheimer’s Disease Knowledge Scale, Negative Interaction and Satisfaction with Social Support, Revised Memory and Behavior Problems Checklist, Mindful Attention Awareness Scale, Quality of Life–Alzheimer’s Disease, Kingstone Caregiver Stress Scale, Interpersonal Reactivity Index, and Dyadic Relationship Scale. Table 1 describes each measure used. Sociodemographic information is collected at the screening.

**Table 1:** Description of the assessments used to assess changes in key outcomes.

Questionnaire^a^		Subdomain	Cronbach *α*	*R*	Description
Sociodemographics (specific to study)		N/A^b^	N/A	N/A	Data collected include age, level of education, race and ethnicity, marital status, occupation, and years providing care [42]
**Proximal (secondary) outcomes**					
	Revised Scale for Caregiving Self-Efficacy	Controlling unhelpful thoughts	.800 to .850	0.790	Assesses the confidence of caregivers of chronic individuals to cope with the care recipient’s demands and challenges. Selected subdomains focus on efficacy for managing distressful thoughts and managing disruptive behavior [43]
	Revised Scale for Caregiving Self-Efficacy	Disruptive behavior	.800 to .850	0.700	Assesses the confidence of caregivers of chronic individuals to cope with the care recipient’s demands and challenges. Selected subdomains focus on efficacy for managing distressful thoughts and managing disruptive behavior [43]
	Preparedness for Caregiving Scale	N/A	.700 to .800	—^c^	Gauges the ability to perform as a caregiver [44]
	Problem-Solving Self-Efficacy Scale	N/A	.830	0.683	Evaluates the caregiver’s confidence to perform a skill and solve a problem [45]
	Alzheimer’s Disease Knowledge Scale	N/A	.710	0.810	Tests knowledge of Alzheimer's disease among the general population and health providers [46]
	Negative Interaction and Satisfaction with Social Support	Negative interaction	.873	—	Assesses social support by using 10 items to measure 3 domains of support: received support, satisfaction with support, and negative interactions of support [25,47,48]
	Negative Interaction and Satisfaction with Social Support	Satisfaction with support	.878	—	Assesses social support by using 10 items to measure 3 domains of support: received support, satisfaction with support, and negative interactions of support [25,47,48]
	Revised Memory and Behavior Problems Checklist	N/A	.840 to .900	—	Evaluates differences in problem behaviors of care recipient and how much it bothered the caregiver [49]
	Mindful Attention Awareness Scale	N/A	.800 to .900	—	Assesses mindfulness awareness though a 15-item questionnaire: how attention and sensitive awareness happens while observing the surroundings [50]
**Distal (primary) outcomes**					
	Quality of Life–Alzheimer’s Disease	N/A	.860	0.100 to 0.650	Measures physical health, mental health, social and financial domains, and overall quality of life [51,52]
	Kingstone Caregiver Stress Scale	N/A	.880	0.880	Monitors stress over time in 3 domains: caregiving, family, and financial issues [53]
	Interpersonal Reactivity Index	N/A	.710 to .770	0.610 to 0.710	Evaluates on 4 subscales how the person reacts when observing others’ experiences [54,55]
	Dyadic Relationship Scale	N/A	.740 to .770	—	Assesses positive and negative aspects of the family relationship [56]

^a^All scales are evaluated before and after the intervention, except for demographics (data gathered during the screening and before the assessment).

^b^N/A: not applicable.

^c^Not available.

### Postintervention Qualitative Interviews

After the 4-week intervention and postintervention assessment are completed, an individual postintervention Zoom interview with a research team member (other than the interventionist) gathers additional insight into the usefulness of the intervention and its materials, such as the VR experience, mindfulness activities and apps, and content of the sessions. Examples of questions include “What are your thoughts on the VR experience?” “How did the experience make you feel?” “How useful do you think the communication component is?” “How useful do you think the mindfulness component is?” “How often did you use it?” “How interesting is it for you?”

### Study Protocol

#### Recruitment and Retention

Nonprobability purposive and snowball sampling is used to recruit the maximum number of participants. Purposive sampling allows for selecting caregivers of people with dementia who meet the aforementioned selection criteria [57]. In addition, participants are asked to refer other people they know who are in the same situation to participate in the study [58].

The recruitment strategy consists of advertising the intervention via social media platforms such as Facebook, Twitter, Instagram, and TikTok. In addition, flyers and related project information are shared with close professional contacts and other providers at community-based organizations such as the local branch of the Alzheimer’s Association and local area agencies on aging, including through invitations to participate sent via email.

To encourage participants to join and complete the workshop, we focus on the following strategies: (1) once a participant contacts the team inquiring about the program, the research team texts, emails, or calls the participants via telephone or videoconference to establish a relationship and offer the opportunity to ask questions before enrolling; (2) provide incentives (eGift cards) after the completion of the pre-post assessments; (3) offer different options of mindfulness and relaxation apps for participants to use; and (4) provide a VR headset compatible with the participant’s mobile phone.

#### Screening

Trained research staff screen participants to determine eligibility via telephone call or Zoom videoconference. Eligible and interested participants complete a second short survey about their fitness to use the VR headset compatible with their mobile phone. This short questionnaire helps to determine whether the participant can safely use VR without physical side effects during the intervention sessions. The questions include “Do you experience motion or car sickness?” “Do you have any conditions where flashing or intense light might affect you, such as epilepsy, migraines, unexplained seizures, recent concussions, or light sensitivity?” If participants answer positively to even one of the questions, they will be deemed noneligible for VR use and asked to visualize the experience through their laptop computers (semi-immersive VR) instead of using the VR headset to avoid potential side effects during the intervention sessions. The questionnaire was determined in coordination with the university institutional review board.

#### Study Enrollment and Consent

To enroll in the program, eligible participants sign the consent form via REDCap (Research Electronic Data Capture; Vanderbilt University), a Health Insurance Portability and Accountability Act–compliant web-based secure tool to capture and manage research data [56]. The consent form is sent to the participant’s preferred email address. In addition, a telephone number is provided to participants in case they need further information or assistance while signing the consent form.

#### Follow-up Assessment Procedures

Enrolled participants fill in the web-based preassessment survey within 2 weeks before the first day of the intervention sessions via REDCap. Once this survey is completed, participants receive a VR headset compatible with their mobile phone if they are eligible for a VR headset based on the screening about VR tolerance, a binder with the materials, and options for mindfulness apps. As part of the screening process, we identified those who were unable to use the headset because of cybersickness. During the intervention, participants are split into 2 groups: VR and semi-immersive VR (ie, unable to use the headset). However, there is no difference in what the 2 subgroups of participants observe during the VR experience, and the VR experience lasts exactly the same amount of time for both groups during the intervention. Thus, the only difference is during the visualization mode of the VR experience to avoid cybersickness symptoms. As this is a feasibility and acceptability study, we gather feedback about each participant’s experience and will be able to comment on differences between the groups as a result. We will explore differences in outcomes across the groups, but as an acceptability and feasibility trial, the study may not have the power to detect differences.

After the intervention, participants fill in the postassessment survey and meet with trained research staff (other than the interventionists) for the individual Zoom interview within 2 weeks after the last web-based check-in. eGift cards are electronically sent to participants who complete each assessment.

### Data Analysis

Data analysis will follow the embedded design approach, a type of mixed methods approach, also known as intervention mixed methods framework [59,60]. The study blends elements of quantitative and qualitative research approaches to gain a deeper understanding of a phenomenon—in this case, the experimental intervention. Equal weight will be given to both components because the qualitative results will help to interpret the quantitative results, describe the participants’ experiences with the intervention, and refine the intervention for future implementation based on the data gathered [59,61].

### Sample Size Considerations

The study objective was to evaluate the feasibility and acceptability of an ICT-delivered intervention for caregivers using a VR component, and 20 caregivers were recruited to date to provide feedback on the VR experience as well as the usefulness of the intervention content. Although the focus is on feasibility and acceptability, the sample will provide us with the opportunity to gather feedback on the project’s pre- and postintervention assessments as well as gather preliminary data on changes in key outcomes. The findings will guide the implementation of lessons learned from the project for adaptation of this intervention or the development of new technologically enhanced interventions in the future.

### Quantitative Data Analysis Strategies

Variable-oriented data will be entered and analyzed using SPSS software (version 23.0; IBM Corp). Descriptive statistics will help to identify distributions (means and frequencies) for sociodemographic information and feasibility outcomes (perception of benefits and satisfaction survey). Paired 2-tailed *t* tests will be used to explore changes between the pre- and postintervention assessments. Given the focus on feasibility and acceptability, the *P* value for statistical significance will be established at *α*<.05 and 95% CI. Cohen *d*will also be computed to describe the magnitude of each mean change to estimate power for future interventions [61].

### Qualitative Data Analysis

Directed content analysis, which allows for the combining of multiple techniques, will be used to analyze data regarding participant perspectives [62]. Individual postintervention Zoom interviews will gather participants’ perceptions of the intervention’s critical components, such as the usefulness of including VR or the amount of time devoted to each session. Participants’ viewpoints will be audio recorded and transcribed using the Zoom transcription function.

The qualitative analysis will consist of (1) information complementing the quantitative data and (2) thematic analysis to unveil and elaborate on components of the intervention such as participants’ views of the VR experience. For data analysis and interpretation, the analytic approach will be based on the indications for coding provided by Miles et al [63], consisting of a first and second cycle of analysis of the transcriptions.

### Data Analysis Steps

#### Overview

The first step of data analysis will be the creation of a codebook with codes and definitions. A list of provisional initial codes will be derived from the research questions and other variables, such as inquiries about the different psychoeducational skill-building components. All the codes will have clear operational definitions.

The anticipated approach for the first cycle of coding will correspond to descriptive coding (1-word summaries), in vivo coding (words used by participants), emotion coding (feelings), evaluation coding (judgments about programs), and theming the data (a phrase that identifies a unit of data). The goal is to create a level 1 code with meaningful units. The first coding cycle will help to identify expected deductive codes (eg, *VR views, communication, challenging behavior, understanding dementia,* and *areas for improvement*). In addition, after a thorough and careful analysis of the data, inductive codes could potentially be identified; therefore, pattern codes will be extracted from the first cycle.

The second cycle of coding will consist of summarizing segments of data using pattern coding, grouping those summaries into a small number of themes (*focused coding*). Codes will be organized in an Excel spreadsheet (Microsoft Corp) using the following format: label used for each code or category, description of the concept or theme the code refers to, samples in the text, notes on how the code relates to others, and quotes. Once data saturation is reached, and the table is populated with all the codes obtained from the interviews, the next step will be to create a tree diagram. The tree diagram will include the main code, subcodes, a second level of subcodes when needed, and quotes that refer to the specific code or subcode. The tree diagram will help to organize and visualize the data, collapsing codes, and explain the relationships among the identified codes. This approach will facilitate interpreting and capturing the main essence of the caregivers’ experiences with the intervention, the different components, what they like, suggestions, and other themes [62]. Two team members will revise the downloaded transcriptions to verify the transcription and data analysis accuracy. In case of discordance, a third researcher will intervene. Excel will be a supporting tool to help organize and display the data.

#### Issues of Trustworthiness

The selected theoretical framework will guide the data analysis process and enable us to draw conclusions. Credibility, dependability, confirmability, transferability, and application will be assessed to ensure trustworthiness. This process will ensure that potential biases affecting the study’s design, implementation, and analysis are diminished and affirm that the conclusions are of high quality [58,63,64].

#### Credibility

To ensure methodological validity (credibility) of the study, we will (1) involve different researchers during the research process, including data analysis, synthesis, and interpretation of the results; (2) revise and discuss the findings with colleagues; and (3) build a logical chain of evidence and present data using illustrations plotting tentative relationships when needed [58,63,64].

#### Dependability

All the procedures will be documented to demonstrate the consistent use of codes and categories to ensure dependability. The involved colleagues will maintain a diary with their data analysis memos and steps to enhance transparency. Finally, a monitoring plan, including a moderator guide, will be created to help junior researchers interpret data [63,65,66].

#### Confirmability

To assess confirmability, memos and transcripts can offer a unique opportunity for the reader to evaluate the findings. In this case, a transcript segment will be facilitated in the form of a figure with the coding process to offer visible evidence of the process followed [58,65].

#### Transferability

The findings will be presented with a rich description of the sample and context, a consistent summary of the findings, and suggestions for new environments to broaden the perspective to ensure transferability [58,59,63].

#### Application

The data obtained will reach consumers via scientific publications, poster presentations, and oral communications. The findings can bring awareness to policy makers and promote further research [66].

## Results

Recruitment began in January 2022, with 20 caregivers across the United States recruited to date. However, recruitment and data collection are still ongoing, and the results are expected to be submitted for publication in the second half of 2023. Preliminary data and feedback from participants support the feasibility and acceptability of the project. All the participants enrolled to date have completed the workshop as well as the pre- and postintervention assessments, including the individual Zoom interview.

## Discussion

### Strengths and Limitations

*Through Alzheimer’s Eyes* is a 4-week multicomponent intervention that includes a VR experience in every session. The intervention is designed to decrease the stress and distress of family caregivers of people with memory challenges by improving communication, behavioral management skills, management of unhelpful thoughts, and mindfulness.

A strength of *Through Alzheimer’s Eyes* is that it combines VR experiences with different skills-driven components to help caregivers of people with ADRD. VR provides a valuable eye-opening opportunity to experience what it is like to have Alzheimer's disease. The intervention is guided by 2 robust theories: SCT and CBT; this increases the possibility of having a positive impact on participants’ behaviors. In addition, the intervention is delivered via Zoom, enhancing the acceptability and feasibility of the intervention because participants can connect from their preferred private location via mobile phone or laptop computer with an internet connection. Connecting from home helps to resolve stressors that arise from in-person interventions (eg, finding respite, transportation, parking fees, and work schedules). This ICT-delivered intervention presents the advantage of reaching individuals living far from the intervention location and allowing caregivers to participate while remaining close to their care recipient, avoiding the hassle of looking for paid caregivers [67-69]. Potential limitations include technical difficulties that can be encountered while using the VR headsets and Zoom, which could affect the timing and flow of the session.

The intervention’s novelty includes using VR and connecting remotely to help caregivers cope with stressful situations and better understand ADRD, as well as improve communication skills, manage unhelpful thinking, and increase confidence when providing care. The analysis and synthesis of the results will guide future research by providing information about VR use for learning purposes and promoting behavior change among caregivers of people with dementia.

### Conclusions

This innovative study merged technology with psychoeducational and skill-building interventions, addressing a gap in the existing research literature. Interventions using ICTs such as VR present unique advantages [70]. In particular, VR brings the user to a quasi-naturalistic real-life situation where, in a safe environment, they can experience having Alzheimer's disease to help them better understand their loved one and provide holistic care. In addition, ICT-delivered interventions can provide cost savings and be more affordable than traditional in-person interventions [69,71]. By including VR and promoting an ICT-delivered intervention, we aim to reduce the number of caregivers who experience diminished well-being and provide the opportunity for caregivers to better care for their loved ones and themselves while ensuring that their own needs are met [72].

Implications for public health research and health care professionals’ education and practice include the opportunity to use technology combined with skill building to (1) broaden awareness of the need to care for the caregiver, (2) advance actions in health care practice that move toward treating the caregiver and care recipient dyad and not focus only on the care recipient’s treatment and health care goals, (3) create new training and related resources to promote healthy aging in place at a lower cost to people with ADRD and their family caregivers, (4) offer VR experiences about having Alzheimer's disease to foster understanding for students and professionals serving the older population, (5) reinforce interdisciplinary education and practice to help caregivers better navigate systems when seeking resources, and (6) enhance partnerships among researchers, health systems, and not-for-profit organizations to embed evidence-based technologically enhanced caregiver interventions across providers to fill gaps and more widely distribute resources for caregiving training. If the results are beneficial, they will provide a basis for a future intervention on a larger scale, including a comparison group. A comparison group will help to draw more robust conclusions regarding changes in key outcomes of interest. Improved intervention content based on participant feedback and translation of the lessons learned through piloting this technologically enhanced intervention will also assist in adapting interventions that help caregivers of patients with other chronic conditions to stay healthier and more interpersonally connected. Future studies will offer the sessions in other languages, such as Spanish, to reach the growing Hispanic and Latino communities and bridge health care inequalities. Technologically enhanced interventions hold promise as a tool for training caregivers that can be expanded in the future to serve a wider community.

## Appendix

Multimedia Appendix 1 Descriptions of the relationships among the constructs, intervention activities, and outcomes that affect each key social cognitive theory and cognitive behavioral therapy construct.

## Abbreviations

ADRD: Alzheimer's disease and related dementias
CBT: cognitive behavioral therapy
ICT: information and communication technology
REDCap: Research Electronic Data Capture
SCT: social cognitive theory
VR: virtual reality

## Data Availability

The data sets generated and analyzed during this study are not publicly available because of ongoing enrollment and future data analyses. However, data generated and analyzed as part of this study will be published in a peer-reviewed journal.
