# Supplementary material for: Behind the Scenes of a Technologically Enhanced Intervention for Caregivers of People With Dementia: Protocol for a Feasibility and Acceptability Study
Source: JMIR Res Protoc. 2023 Mar 31;12:e42655. doi: 10.2196/42655 (PMC10131762; doi:10.2196/42655)
Supplement: Multimedia Appendix 1 [file resprot_v12i1e42655_app1.docx]

**Table 1.** Descriptions of the relationships among the constructs, intervention activities, and outcomes used that affect in each key social cognitive theory (SCT) and cognitive behavioral therapy (CBT) construct.

| Key theoretical construct and description | | Construct subcategories and description | Intervention activity | How the theoretical construct is reflected in the intervention components | Outcome (enhanced/improved) |
| --- | --- | --- | --- | --- | --- |
| **SCT** | | | | | |
|  | Behavioral capability: knowing what to do in different challenging circumstances and acquiring essential knowledge and skills for providing better care to their loved one and themselves | N/A^a^ | Discussions; mini-lecture; VR^b^ experience | - During the 4 weeks, the caregivers participate in the following activities:   1. Lecture about dementia, communication, controlling upsetting thoughts, and responding to disruptive behavior   2. VR experience with emphasis depending on the weekly topic   3. Discussion to reinforce knowledge regarding the weekly topic - Participants have access to resources, written text with the information, and links to reliable websites | - Knowledge of dementia care, symptoms, and behavioral manifestations - Knowledge of how to communicate more effectively - Knowledge of how to control upsetting thoughts - Knowledge of available resources |
|  | Outcome expectations: outcomes of the behavior/actions of engaging in the intervention and health consequences of the intervention, such as reduced stress and improved quality of life | N/A | Mini-lecture; discussions; VR experience; mindfulness exercises | - Discussions and materials are directed toward enhancing the relationship between the dyad (caregiver and care recipient), their well-being, decreasing caregiver stress using effective communication, understanding the disease, managing negative thoughts, and improving problem-solving techniques | - Improved overall relationship with the care recipient - Improved overall quality of life - Diminished overall stress levels - Improved understanding of dementia - Better management of negative thoughts - Better problem-solving skills |
|  | Self-efficacy: the individual’s belief in their capacity to execute an action or to perform | Mastery experience: previous performance and knowledge acquired | VR experience; activities; practice; mini-lecture; individual discussion sessions | - During the 4 weeks, to gain mastery experience, the training provides instructions and practice opportunities in the form of the following activities:   1. Weekly lectures with topics concerning Alzheimer disease, communication, controlling unhelpful thoughts, and problem-solving   2. Discussion sessions to share tips among participants and participants’ experiences of what works for them   3. VR visualization centered on the weekly topic to understand the lived experience, gain knowledge of dementia, and acquire a new experience   4. Practice communication, thought management, and problem-solving via activities or homework   5. Practice sessions and homework to reinforce knowledge | - Improved communication skills, techniques for controlling unhelpful thoughts, and problem-solving - Diminished overall stress levels - Improved dementia knowledge |
|  |  | Vicarious experience: gaining experience through others, imitation, and stories | Practice; discussions | - Learning by observing others and modeling to lead by example - The capacity to learn by observation enables people to acquire rules for generating and regulating behavioral patterns without having to form them gradually by tedious trial and error - Mastering skills through the aid of modeling | - Confidence when dealing with challenging behavior - Improved ability to communicate effectively - Diminished overall stress - Knowledge of dementia |
|  |  | Verbal persuasion: telling participants that they are capable; verbal encouragement | Discussions; practice; individual sessions | - Discussions during the session offer the opportunity and safe environment to provide feedback to the participants and encourage participants between each other - Out-of-session support (Zoom *office hours*) will facilitate support for the participant | - Confidence when providing care, resolving challenging situations, ability to reframe thoughts and when using communication skills, and managing stress |
|  |  | Emotional arousal: emotional states affecting confidence (ie, anxiety, stress, mood, and arousal) | Practice; activities; mindfulness exercises | - Discussions, practicing the skills, and VR experience will reinforce the idea that challenging situations can be resolved calmly | - Confidence in problem-solving and responding to disruptive behavior and stress |
|  | Social influence/social support: social reinforcement to improve motivation and behavior | Emotional support: empathy, love, trust, and care | Discussions; practice; individual sessions | - Weekly discussion sessions to share tips and experiences among participants and encourage each other to try a different approach when solving challenging situations - Reinforce participation in activities with other people - Respite solutions - Zoom *office hours* session with interventionist to provide encouragement and support | - Improved confidence when performing care activities, reacting to challenging behavior, communicating with their loved one, and controlling unhelpful thoughts - Enhanced caregiver emotional and social support and support with caregiving activities - Increased empathy toward their care recipient and different situations that might arise |
|  |  | Appraisal support: constructive feedback and affirmation | Individual sessions; discussions; practice | - Discussions and practice provide the opportunity to share suggestions, reaffirm knowledge, and provide feedback about challenging situations and how to tackle them - Zoom *office hours* session with interventionist provides encouragement and support to the participant | - Improved confidence when performing care activities, reacting to challenging behavior, communicating with their loved one, and controlling unhelpful thoughts - Empathy toward the care recipient and persons with dementia - Enhanced caregiver emotional and social support as well as support with different caregiving activities |
|  |  | Informational support: advice, suggestion, and information | Materials; individual sessions; discussions | - Provide community resources and explain how to access them - Zoom *office hours* session to support and tailor the resources needed | - Improved knowledge and satisfaction with support and resources available for caregivers |
| **Skill-building model (CBT)** | | | | | |
|  | Redirecting negative thoughts (cognitive restructuring) | Behavioral outcome: if thoughts can be changed, behavior will change (thoughts mediate behavior) | Discussions; activities | - In week 4, participants learn how to manage negative or unhelpful thoughts. The activities and discussion include the following:   1. Listing 2 unhelpful thoughts to change or manage   2. Assessment of the thought(s): frequency, what would help, attempts to control unhelpful thoughts   3. Develop an action plan   4. Set a goal   5. Celebrate accomplishments   6. Homework | - Enhanced abilities and skills for controlling and redirecting negative thoughts |
|  | Problem-solving: approach of choice that helps to solve many problems | N/A | Activities; practice; VR experience | - In week 3, the participant learns the problem-solving approach. The activities and discussion focus on finding solutions to different problems that arise often when caring for a person with Alzheimer disease | - Enhanced abilities and skills for finding solutions to problems |
